# Are surveillance response systems enough to effectively combat and contain the Ebola outbreak?

**DOI:** 10.1186/2049-9957-4-7

**Published:** 2015-01-09

**Authors:** Viroj Wiwanitkit, Ernest Tambo, Emmanuel Chidiebere Ugwu, Jeane Yonkeu Ngogang, Xiao-Nong Zhou

**Affiliations:** Surin Rajabhat University, Bangkok, Thailand; Joseph Ayo Babalola University, Osogbo, Nigeria; Sydney Brenner Institute for Molecular Bioscience, Wits 21st Century Institute, Faculty of Health Sciences, University of the Witwatersrand, Johannesburg, South Africa; Department of Biochemistry, Center for Sustainable Malaria Control, Faculty of Natural and Agricultural Sciences, University of Pretoria, Pretoria, South Africa; Department of Human Biochemistry, Faculty of Basic Medical Sciences, Nnamdi Azikiwe University, Awka, Nnewi Campus, Nigeria; Département de Biochimie, Faculté des Sciences Biomédicales, Université de Yaoundé, Yaoundé, République du Cameroun; National Institute of Parasitic Diseases, Chinese Center for Disease Control and Prevention, and the WHO Collaborating Centre on Malaria, Schistosomiasis and Filariasis, Shanghai, 200025 PR China

**Keywords:** Ebola, Outbreak, Surveillance response systems, Africa

## Abstract

The epidemic of the Ebola virus infection in West Africa in 2014 has become a worldwide concern. Due to the nature of the disease, which has an extremely high mortality potential, this outbreak has received much attention from researchers and public health workers. An article entitled "Need of surveillance response systems to combat Ebola outbreaks and other emerging infectious diseases in African countries," published in the journal *Infectious Diseases of Poverty* in August 2014, concluded that a good surveillance system to monitor disease transmission dynamics is essential and needs to be implemented to combat the outbreak. Issues regarding the limitation of the passive surveillance system have been raised by Professor Viroj Wiwanitkit, who emphasizes the need for an active disease detection system such as mass screening in this letter to editor. The different function between passive and active surveillance system in combating the disease outbreak has been agreed upon by Ernest Tambo et al. There have also been discussions between Wiwanitkit and Tambo et al. on the following issues: (i) the extreme resource limitations in outbreak areas, (ii) new technology to improve the available systems. Further recommendations echoed in this letter to editor by Wiwanitkit, who outlined the research priorities on the development of appropriate combined disease monitoring systems and good policy to allocate available tools and technology in resource-limited settings for epidemic scenarios. The journal’s editor, Professor Xiao-Nong Zhou, has therefore collated all parts of these discussions between authors in this letter to editor paper, in order to further promote research on a combined active and passive system to combat the present extending Ebola outbreak.

**Electronic supplementary material:**

The online version of this article (doi:10.1186/2049-9957-4-7) contains supplementary material, which is available to authorized users.

## Multilingual abstracts

Please see Additional file 1 for translations of the abstract into the six official working languages of the United Nations.

## Background

The epidemic of the Ebola virus disease (EVD) in West Africa in 2014 continues to present a global concern due to its extremely high mortality potential, and short- and long-term regional and international consequences. The West African EVD outbreak has received much attention from researchers, humanitarian organizations, and international agencies, as well as from public health workers and communities [1, 2]. An article entitled "Need of surveillance response systems to combat Ebola outbreaks and other emerging infectious diseases in African countries," published in the journal *Infectious Diseases of Poverty* in August 2014 [3], concluded that implementation of an effective surveillance response system with early warning alert mechanisms and the ability to measure transmission dynamics is essential to monitor disease epidemics, and consequently prevent and combat this outbreak and the future emerging epidemic. The article also discussed that an Ebola immunization program and travel medicine initiatives are needed. Issues regarding the limitation of the passive surveillance system have been raised by Viroj Wiwanitkit in this letter to editor, who emphasizes the need for an active disease detection system such as massive population screening and other interventions. This idea has been agreed upon by Ernest Tambo et al. There have also been discussions between Wiwanitkit and Tambo et al. in this letter to editor on the extreme resource limitations in outbreak areas. Maximizing the contemporary scientific and technological benefits can greatly improve and strengthen the availability of and accessibility to the much-needed tools and systems, including early detection, early warning alert, planning, and emergency responses.

This was further echoed by Professor Wiwanitkit, in this letter on the previous publication by Tambo et al. [3], who outlined the research priorities for the development of an appropriate combined disease monitoring system along with good policy to allocate the most effective available tools, and enhance technology and information communication in resource-limited settings in different epidemic scenarios. The journal’s editor (Professor Xiao-Nong Zhou) thus combined all of these discussions in one paper, in order to further promote research on moving from a passive surveillance system to an active and integrated surveillance response system that will combat and contain the present extending and future outbreak. This article looks at the importance and limitations of each system and calls for the need to foster more research and set priorities aimed towards novel comprehensive integrated surveillance response systems. These systems should be able to collate effective and reliable data that can support evidence-based decisions and policy and guide emergency responses, in addition to serving as early warning and prompt response systems in the prevention, control, and containment of Ebola and future emerging diseases.

### Combating Ebola outbreaks: other options besides surveillance response systems

Viroj Wiwanitkit believes that Ebola outbreaks can be combated by other means besides surveillance response systems. He quotes what Tambo et al. reported in the aforementioned article: "Understanding the unending risks of transmission dynamics and resurgence is essential in implementing rapid effective response interventions tailored to specific local settings and contexts" [3].

The emerging Ebola virus is a serious regional and international consideration with short- and long-term crisis ramifications and consequences on the social, health, and economic statuses of nations. Several attempts have been made to stem out this new emerging viral infection since the first outbreak occurred, however, the control and containment of the disease in West Africa has not been successful. A major concern is its high mortality rate and fate of survivors, especially the thousands of orphans who have been abandoned, as well as the compounded issue of years of political unrest that has been worsened by the Ebola crisis. Up to date, more than 450 health workers have contacted the virus from patients, with over 150 deaths reported [4]. To successfully control the Ebola outbreak, surveillance response systems are undoubtedly important tools. However, whether they are effective enough on their own is questionable. There is a vital need to enhance and strengthen community health education and promotion, local capacity building, and knowledge of EVD and other epidemics, and implement safety measures for vulnerable communities.

In fact, to successfully control and contain the outbreak, urgent and early emergency response preparedness and planning is required. Raabea and Borcherta noted that, "effective identification and isolation of cases, timely contact tracing and monitoring, proper usage of personal protection gear by health workers, and safely conducted burials" are essential for success [5]. Surveillance response systems, which are generally used for the management of an outbreak, can be useful, however, they are a passive method as they simply gather data about an already existing infection. Although new computational technology might be applied to predict possible new foci of disease, it is not an actual finding but a computational prediction. Additional active method should be applied to increase the effectiveness of disease control. The use of active disease surveillance such as mass screening is suggested [6]. It should be noted that the asymptomatic Ebola virus infection is possible and might be the reservoir source of infection [7]. Finally, along with standard available techniques, the medical community urgently needs to develop new drugs and vaccines for confronting the possible pandemic of this new emerging disease.

### Ebola spreading in the poor settings of West Africa with inexistent or inadequate healthcare systems

Ernest Tambo, Emmanuel Ugwu, and Jeane Ngogang highlighted that Ebola persistence and spread is due to the poor or inadequate healthcare systems, the impact of mining and conflict events, high levels of illiteracy, and rampant medical, community and environmental capacity building in most of the Ebola affected countries in West Africa, as compared to developed countries. The authors thanked Wiwanitkit for his remarks on how the surveillance and response system to combat the Ebola outbreaks can be improved. As Tambo, who has more than 10 years experience working in West Africa, and his team [8] clearly stated, it is an essential and crucial step to implement surveillance and response preparedness and actions effectively and in a timely manner. Similarly, in other African countries with little passive and periodic surveillance and inherent structural deficiencies, high levels of rural illiteracy and ignorance on epidemics, persistent cultural practices and myths, rumors and the inability of the governments to debunk them, a lack of aggressive public health awareness and community outreach with appropriate health education, fragile healthcare service delivery, maternal-child mortality, slow responses, inappropriate use of protective device and disinfectants, health education media (TV, radio, mobile texts), and limited government funds and reliance on intentional donors such as Global Fund to Fight HIV/AIDS, Tuberculosis and Malaria, it is difficult to contain the spread of Ebola without local surveillance response systems [8].

Thus, in line with Wiwanitkit’s concerns, active and integrated surveillance response systems should be developed with networks of community/environmental workers, which include early warning, early diagnosis, quick reporting, case finding, case tracking and investigation, and prompt appropriate measures including treatment to stop the transmission. In addition, it is also necessary to foster more efforts in new viral diagnostic and drug/vaccine discovery, as well as innovative measures based on local contexts. Tambo et al. also outline that components of new technology development are needed to improve the sensitivity of surveillance response systems [1]. It is clear that inexistent or inadequate healthcare systems and bottlenecks are compounded by other inequalities (majorly illiteracy and multi-dimensional poverty index) in remote African communities and urban settings [8]. It is these local settings with a lack of adequate interventions that play the most important role in the spread of the Ebola virus in the human population [9]. Please refer to Tambo et al.’s article to understand the current Ebola crisis and what the next steps should be [1]. Tambo et al. will be pleased to work with Wiwanitkit on the topic of proper surveillance response systems design and share lessons and expertise about Ebola and H7N9.

### How to deal with the problem in resource-limited settings and future research priorities

Wiwanitkit responded that dealing with the problem in resource-limited settings requires local and context evidence in setting health agendas and research priorities. Good planning and effective allocation of resources, which are limited, has to rapidly done [10]. The education of local medical personnel to have the correct knowledge aimed at disease prevention and management is required [10].

As argued by Tambo et al., the present passive disease surveillance system to combat the Ebola virus epidemic in Africa appears to not be sufficient as the infection is spreading rapidly. In addition, the available disease surveillance systems in many African countries might not be fully effective [1]. Theoretical political commitment, fancy health investment, and human resource development are the major obstacles in building reliable health systems and surveillance responses for disease outbreaks in the region. What is also of interest is that although the disease has been continuously spreading, there are only a few available data on clinical features and epidemiological information [10]. Limited publications are available on these issues [11, 12].

An extreme limitation of resources is a basic fact for most infectious diseases’ endemicity and epidemicity in Africa. The problem of disease control in this continent is related to poverty [13]. The present Ebola virus crisis is the greatest challenge to local disease surveillance and control agencies [14]. As noted by Smith et al., "more support needs to be given to core coordinating capacity in resource-poor contexts" [15]. Grants from many countries around the world to combat the problem are a good sign of collaboration. Without a doubt, research and development of new technologies to manage the problem is also useful. As noted by Tambo et al., "improving the sensitivity of the surveillance response system" might be another practical approach for the present crisis [8]. The development of appropriate combined disease monitoring systems, incorporating both passive and active approaches, might be the answer for managing the rapid expansion of the infection. Electronic integrated systems should also be considered and implemented [16].

Finally, the local national and regional public health policy seems to be an important determinant. A good policy that allocates the available tools and technologies in resource-limited settings in the epidemic scenario is required. The recent international meeting of the relevant West African health ministers to talk about the Ebola crisis is a good sign and gives hope for successful epidemic control [11]. The following research priorities are put forward to respond to the Ebola outbreaks in Africa:

1. Political research to find ways for a synchronized international collaboration to combat the outbreak;
2. Local development of diagnostic tools to help active case search; and
3. Development of a multidisciplinary computational approach/system and a geographic information system (GIS)-based prediction model for closed, up-to-date outbreak monitoring.

## Electronic supplementary material

Additional file 1: **Multilingual abstracts in the six official working languages of the United Nations.** (PDF 233 KB)
