# Supplementary material for: Are surveillance response systems enough to effectively combat and contain the Ebola outbreak?
Source: Infect Dis Poverty. 2015 Jan 9;4(1):7. doi: 10.1186/2049-9957-4-7 (PMC4322436; doi:10.1186/2049-9957-4-7)
Supplement: Supplementary file 1 — Additional file 1: Multilingual abstracts in the six official working languages of the United Nations. (PDF 233 KB) [file 40249_2014_89_MOESM1_ESM.pdf]

Translation of the abstract into the six official working languages of the United Nations

هل أنظمة الاستجابة للمراقبة كافية للمكافحة الفعّالة واحتواء تفشي مرض الإيبولا؟

فيروج ويوانيتكيت، إرنيست تامبو، إمانويل تشيديبير أوجوو، جين يونكو نجوجانج، كسايو-نونج زهو.

ملخص

أصبح وباء عدوى فيروس الإيبولا في غرب أفريقيا عام 2014 مصدر قلق عالمي. ونظراً لطبيعة هذا المرض الذي يتسم بنسبة وفيات عالية للغاية، فقد حظي على اهتمام كبير من الباحثين والعاملين في مجال الصحة العامة. وخلص مقال بعنوان "الحاجة لأنظمة استجابة للمراقبة لمكافحة تفشي مرض الإيبولا والأمراض المعدية الأخرى الناشئة في البلدان الأفريقية" في دورية FΘ ŭ ŭ ŭ ĭ في عدد أغسطس 2014، أن وجود نظام مراقبة جيد لمراقبة ديناميكيات انتقال المرض لهو أمر ضروري ويجب أن يفعّل لمكافحة تفشي الوباء. وقد أثيرت مواضيع متعلقة بالحد من نظام المراقبة السلبي في رسالة للمحرر من قبل البروفسور فيروج ويوانيتكيت والذي أكد فيها على الحاجة لنظام فعّال للكشف عن الأمراض مثل الفحص الشامل. وقد اتفق إرنست تامبو وآخرون على الوظائف المختلفة لنظام المراقبة السلبية والإيجابية لمكافحة تفشي المرض. وكانت هناك أيضاّ مناقشات بين ويوانيتكيت وتامبو وآخرين على المواضيع التالية: (أ) المصادر المحدودة جداّ في مناطق تفشي المرض، (ب) تقنية جديدة لتحسين النظم الموجودة. كما وردت توصيات أخرى في رسالة ويوانيتكيت للمحرر حيث أوجز أولويات البحث على تنمية نظم مناسبة لمراقبة المرض مع سياسة جيدة لتوزيع الأدوات المتاحة والتكنولوجية في المناطق ذات المصادر المحدودة في سيناريوهات الوباء. وقد جمع رئيس التحرير البروفسور كسايو-نونج زهو جميع المناقشات التي تمت بين المؤلفين في هذه الرسالة لمواصلة تعزيز الأبحاث في النظم السلبية والإيجابة معاً لمكافحة تفشي مرض الإيبولا الممتد في الوقت الحالي.

Translated from English version into Arabic by T. Catherine Hanna, through

TRANSLATORS
WITHOUT BORDERS

监测响应系统能足够抗击和遏制埃博拉暴发吗？

Viroj Wiwanitkit, Ernest Tambo, Emmanuel Chidiebere Ugwu, Jeane Yonkeu Ngogang, Xiao-Nong Zhou

摘要

2014 年流行于西非埃博拉病毒感染的疫情已受到全球关注。由于该病的高病死率特征，这次暴发使研究人员和公共卫生工作者尤其关注。在 Infectious Diseases of Poverty 杂志上（2014 年 8 月）发表的一篇题为“抗击非洲国家埃博拉暴发和其它新发传染病对监测响应系统的需求”论文中指出，一个良好的监测疾病传播动态的监测系统对实施抗击疾病暴发工作是最基本的需求。而本文中 Viroj Wiwanitkit 分析了被动监测系统在处置这类问题中的局限性，并强调主动监测（如对人群的筛查）的重要性。Ernest Tambo 等认同了关于主动与被动监测在抗击疾病暴发中的不同作用。Wiwanitkit 与 Tambo 等还讨论了如何解决以下两个方面的问题：（i）在暴发区域内资源溃乏的问题，（ii）利用新技术提升现有卫生系统作用的问题。Wiwanitkit 进一步就如何在疾病暴发资源严重不足情况下研发适宜的综合疾病监测系统和合理分配现有工具与技术等研究重点领域提出了建议。本杂志周晓农主编将双方的讨论意见综合成本文，以进一步推动主动监测与被动监测相结合的卫生系统来控制正在播散的埃博拉暴发疫情。

Translated from English version into Chinese by Zhou Xiao-nong, through

Les systèmes de surveillance et d’intervention actuels suffisent-ils pour combattre efficacement et endiguer l’épidémie d’Ebola?

Viroj Wiwanitkit, Ernest Tambo, Emmanuel Chidiebere Ugwu, Jeane Yonkeu Ngogang et Xiao-Nong Zhou

Résumé

L'épidémie d'infection par le virus Ebola qui a sévi en Afrique de l'Ouest en 2014 a préoccupé le monde entier. Le risque de mortalité lié à cette maladie étant très élevé, cette épidémie a focalisé l'attention tant des chercheurs que des intervenants du secteur de la santé publique. Les auteurs de « Need of surveillance response systems to combat Ebola outbreaks and other emerging infectious diseases in African countries » (*Infectious Diseases of Poverty,* août 2014), article portant sur la nécessité des systèmes de surveillance et d'intervention pour la lutte contre les épidémies – notamment celle d'Ebola – en Afrique, ont conclu à la nécessité de la mise en place d'un système de surveillance efficace pour suivre la dynamique de transmission de la maladie. Dans la présente contribution, Wiwanitkit soulève la question des limites des systèmes de surveillance passive, insistant sur la nécessité de mettre en place un système de détection active des maladies, notamment par un dépistage de masse. Tambo et coll. mettent par ailleurs en évidence la complémentarité des systèmes de surveillance passifs et actifs dans la lutte contre l'épidémie. Wiwanitkit et Tambo et coll. soulignent collectivement le manque criant de ressources dans les zones touchées par l'épidémie et le rôle des nouvelles technologies dans l'amélioration des systèmes existants. Wiwanitkit recommande au final de concentrer en priorité les efforts de recherche sur la création de systèmes de surveillance mixtes adaptés et de mettre en place des politiques visant à distribuer les outils et technologies disponibles dans les régions dépourvues des ressources nécessaires à la gestion de scénarios épidémiologiques. Zhou collige et présente ainsi les éléments de cette discussion entre les auteurs, afin de promouvoir la recherche visant l'instauration d'un système combinant surveillance active et passive qui soit apte à combattre l'actuelle flambée de virus Ebola.

Translated from English version into French by Isabelle Collombat, through

TRANSLATORS
WITHOUT BORDERS

Достаточно ли эффективны системы реагирования на результаты мониторинга для эффективной борьбы со вспышкой эпидемии Эбола и ее сдерживания?

Вирoдж Виваниткит, Эрнест Тамбо, Эммануэль Чидиебере Угву, Жан Йонкеу Нгоганг, Сяо-Нун Чжоу

Аннотация

Эпидемия вирусной инфекции Эбола в Западной Африке 2014 года вызвала обеспокоенность во всем мире. Из-за характерной для этого заболевания чрезвычайно высокой вероятности летального исхода данная вспышка привлекла пристальное внимание исследователей и работников общественного здравоохранения. В статье под названием "Для борьбы со вспышками Эбола и других формирующихся очагов инфекционных заболеваний в африканских странах необходимы системы реагирования на результаты мониторинга", опубликованной в августе 2014 г. в журнале "Инфекционные болезни бедности", делается вывод о том, что для наблюдения за динамикой распространения болезни существенное значение имеет хорошая система мониторинга и для борьбы со вспышкой следует реализовать ее. Вопросы, касающиеся недостатков системы пассивного мониторинга, поднимает профессор Вирoдж Виваниткит, который в своем читательском письме подчеркивает необходимость активной системы выявления заболевания, такой, как массовый скрининг. Эрнест Тамбо и другие согласны с тем, что функционирование систем пассивного и активного мониторинга при борьбе со вспышкой болезни различно. Виваниткит и Тамбо и другие авторы обсуждают также следующие вопросы: (i) крайнюю ограниченность ресурсов

в областях вспышки, (ii) новую технологию для улучшения уже имеющихся систем. В письме в редакцию Виваниткита отражены и другие рекомендации: так, он очерчивает исследовательские приоритеты в развитии соответствующих комбинированных систем мониторинга заболеваний и правильную политику распределения наличных средств и техники в условиях ограниченных ресурсов при эпидемических сценариях. Ввиду этого редактор журнала профессор Сяо-Нун Чжоу сводит в настоящем обзоре читательских писем все части данного обсуждения разными авторами воедино с целью помочь исследованиям в области комбинированных активных и пассивных систем для борьбы с расширяющейся сейчас вспышкой Эбола.

Translated from English version into Russian by Alexander Somin, through

Son suficientes los sistemas de vigilancia y respuesta para combatir y controlar eficazmente el brote de ébola?

Viroj Wiwanitkit, Ernest Tambo, Emmanuel Chidiebere Ugwu, Jeane Yonkeu Ngogang, Xiao-Nong Zhou

Resumen

La epidemia de infección por el virus del Ébola en África Occidental en 2014 ha pasado a ser un problema a nivel mundial. Debido a la naturaleza de esta enfermedad, que tiene un potencial de mortalidad extremadamente elevado, este brote ha recibido mucha atención de los investigadores y el personal sanitario. En un artículo titulado "Need of surveillance response systems to combat Ebola outbreaks and other emerging infectious diseases in African countries" ("La necesidad de sistemas de vigilancia y respuesta para combatir los brotes de ébola y otras enfermedades infecciosas emergentes en países africanos"), publicado en la revista *Infectious Diseases of Poverty* en agosto de 2014, se concluyó que un buen sistema de vigilancia para monitorear la dinámica de transmisión de la enfermedad es fundamental y debe implementarse para combatir el brote. El profesor Viroj Wiwanitkit ha planteado cuestiones relativas a las limitaciones del sistema de vigilancia pasivo, por lo que hace hincapié en la necesidad de un sistema de detección de enfermedades activo, como el cribado masivo, en una carta al editor. Ernest Tambo *et al* están de acuerdo con la diferencia en las funciones de los sistemas de vigilancia pasivo y activo en el combate contra el brote de la enfermedad. Wiwanitkit y Tambo *et al* también han intercambiado ideas sobre las siguientes cuestiones: (i) las limitaciones de recursos extremas en las áreas del brote, (ii) nuevas tecnologías para mejorar los sistemas existentes. En esta carta al editor, Wiwanitkit también se hizo eco de otras recomendaciones, resumió las prioridades de investigación sobre el desarrollo de sistemas combinados adecuados para el monitoreo de enfermedades y una buena estrategia política para asignar las herramientas y tecnologías disponibles en contextos de recursos limitados para casos de epidemias. El editor de la revista, el profesor Xiao-Nong Zhou, compaginó todas las partes de esos intercambios entre autores en este artículo de cartas al editor, a fin de promover una mayor investigación sobre un sistema activo y pasivo combinado para enfrentar el creciente brote de ébola actual.

Translated from English version into Spanish by Ana Dubra, through
